# WHAT FAMILY CAREGIVERS FEEL UNPREPARED FOR: DIFFERENCES BETWEEN DEMENTIA AND NON-DEMENTIA CAREGIVERS

**DOI:** 10.1093/geroni/igae098.4306

**Published:** 2024-12-31

**Authors:** Roshni Singh, Richard Munoz, Sandra Garcia-Davis, Saanvi Lamba, Stuti Dang, Diana Ruiz, Luci Leykum, Marianne Desir

**Affiliations:** University of Miami, Miami, Florida, United States; South Florida Veteran Affairs Foundation for Research and Education, Miami, Florida, United States; University of Miami, Miami, Florida, United States; Flint Hill School, Oakton, Virginia, United States; Miami VA Healthcare System, Miami, Florida, United States; Miami Veterans Affairs Healthcare System, Miami, Florida, United States; South Texas Veterans Health Care System, Austin, Texas, United States; Miami Veterans Affairs Healthcare System, Miami, Florida, United States

## Abstract

This study identified tasks for which informal caregivers feel insufficiently prepared, stratified by Veterans’ dementia status. Using the Veterans Affairs’ HERO CARE Survey data, we analyzed caregivers’ responses to one open-ended question: “Out of all the tasks that you help the Veteran with, is there anything specific you would like to be better prepared for?” Responses were coded thematically into ten domains and differences in reported domains between caregivers of Veterans with and without dementia were compared. 296 dementia and 426 non-dementia caregivers were included. On average, caregivers were female (87.3%), non-Hispanic white (71.4%), primary caregivers (85.9%), and had provided over five years of care (54.2%). Dementia and non-dementia caregivers were demographically similar except in age (70.9 vs 65.9 years, p< 0.001); spouses (69.1% vs 58.2%, p=0.004); working at-least part-time (18.3% vs 25.9%, p=0.003), and hours of care-provision per week (94.7 vs 74.5, p< 0.001). Preparedness concerns for both dementia and non-dementia caregivers respectively included care-coordination (24.3% vs 25.1%, p=0.81), nursing/medical tasks (19.3% vs 21.6%, p=0.44), advance planning (16.6% vs 15.7%, p=0.77), and assistance with Instrumental Activities of Daily Living (IADLs) (11.5% vs 9.4%, p=0.36) and ADLs (11.5% vs 7.3%, p=0.05). Dementia caregivers expressed significantly more concerns about caregiver self-care (7.4% vs 3.1%, p=0.007), and less about handling emergencies (1.7% vs 4.7%, p=0.03) than non-dementia caregivers. Informal caregivers may need additional training in several domains of care tasks ranging from direct care to service coordination to improve caregiving competency. Dementia caregivers may particularly benefit from learning self-care strategies.

